# Single-cell characterization of the adult male hippocampus suggests a prominent, and cell-type specific, role for *Nrgn* and *Sgk1* in response to a social stressor

**DOI:** 10.1038/s41380-025-03417-y

**Published:** 2026-01-19

**Authors:** Carlo De Donno, Juan Pablo Lopez, Malte D. Luecken, Aron Kos, Elena Brivio, Joeri Bordes, Huanqing Yang, Jan M. Deussing, Mathias V. Schmidt, Fabian J. Theis, Alon Chen

**Affiliations:** 1 Department of Stress Neurobiology and Neurogenetics, Max Planck Institute of Psychiatry, Munich, Germany; 2 Institute of Computational Biology, Helmholtz Zentrum München, German Research Center for Environmental Health, Neuherberg, Germany; 3 Department of Neuroscience, Karolinska Institutet, Stockholm, Sweden; 4 International Max Planck Research School for Translational Psychiatry, Munich, Bavaria 80804 Germany; 5 Department of Brain Sciences, Weizmann Institute of Science, Rehovot, 76100 Israel; 6 Department of Molecular Neuroscience, Weizmann Institute of Science, Rehovot, 76100 Israel; 7 Research Group Neurobiology of Stress Resilience, Max Planck Institute of Psychiatry, Munich, Germany; 8 Molecular Neurogenetics, Max Planck Institute of Psychiatry, 80804 Munich, Germany; 9 School of Computation, Information and Technology, Technical University of Munich, Munich, Germany

**Keywords:** Neuroscience, Molecular biology, Depression

## Abstract

Stress-related psychiatric disorders impact the quality of life of half a billion people around the world. However, our understanding of the molecular mechanisms responsible for stress-response regulation remain unclear. Here, we report the largest and most comprehensive characterization of the adult male mouse hippocampus, under baseline and acute stress condition, using single-cell RNA sequencing. We further used genetically modified knockout lines for the glucocorticoid and mineralocorticoid receptors (GR and MR); two transcription factors which are pivotal regulators of the central stress-response. We found previously unknown, cell-type specific, molecular signatures of a single prolonged social defeat stress response and identified *Nrgn* and *SgK1* as key regulators in stress-responsive glutamatergic neurons, oligodendrocytes, astrocytes, and endothelial cells. Intriguingly, GR or MR deletion, specifically in glutamatergic or GABAergic neurons, led to distinct and cell-type specific transcriptional signatures after stress exposure. This study significantly advances our understanding of the molecular and cellular network underlying the central response to stressful stimuli.

## Introduction

Stress-related psychiatric disorders are a burden for more than 500 million people around the globe [1]. These include illnesses like major depressive disorder, post-traumatic stress disorder and anxiety disorder [2]. Moreover, stress can indirectly lead to health issues induced by dysfunctional behavior [3, 4], such as an increase in alcohol consumption [5] and other addictions [6]. Moreover, levels of stress in the population have increased sharply in recent years due to the increase in external and unpredictable stressors associated with major global events [7–9]. These figures highlight that a maladaptive stress response is becoming one of the main factors leading to a poorer quality of life for many people across gender, ethnicity and age, and that a clear understanding of the mechanisms underpinning the stress response has the potential to improve the life of the affected people.

The response to stress is mediated by complex systems interacting with each other [10]. One of the most important is the hypothalamic-pituitary-adrenal (HPA) axis, a neuroendocrine system which, once activated by a stressor, brings about an increased secretion of glucocorticoid hormones (GCs) [11]. Next to their peripheral actions, GCs affect neural functions by binding to glucocorticoid and mineralocorticoid receptors (GR and MR, respectively). These receptors are ligand-activated transcription factors whose signaling pathways and balance therein have been implicated in the response to stress [12–14]. While GR is present across various regions of the brain, MR is most highly expressed in the hippocampus, making it a region of particular interest for studying the interplay between GR and MR in the regulation of the central stress response. Moreover, the hippocampus is organized along a dorsoventral axis, with both regions contributing to memory, emotional regulation, and stress responsiveness. While the ventral hippocampus has been particularly associated with affective and stress-related processes, the dorsal hippocampus also plays a critical role, especially in glucocorticoid receptor signaling and contextual memory. In this study, we focused on the posterior hippocampus, which includes portions of both dorsal and ventral regions, allowing us to capture a broad transcriptional landscape relevant to stress neurobiology [15].

In this study, we generated a comprehensive single-cell RNA-sequencing (scRNA-seq) catalog and resource for research on the central stress response. We collected cell-type specific gene expression data from the posterior hippocampus (Hipp) of 36 adult male mice, after single prolonged social defeat stress exposure and control conditions. Additionally, to further explore the role of GR and MR in the stress response, we generated and used conditional knockout mouse (cKO) lines for GR and MR, in either glutamatergic or GABAergic neurons of the forebrain (including the hippocampus). In total, we sequenced the transcriptome of 125,896 hippocampal cells. Our experimental design allowed us to characterize the cellular landscape of the adult murine hippocampus, revealing both cell identities and the anatomical location of various clusters of cells. We further identified molecular changes induced by social stress across cell types, and assessed how these changes were affected by the genetic modifications in the GR and MR cKO mice. Notably, we identified a previously unknown molecular signature of the acute stress response in mature oligodendrocytes and a subpopulation of stress-responsive glutamatergic neurons in the. Interestingly, GR or MR deletion, specifically in glutamatergic or GABAergic neurons, led to distinct and cell-type specific transcriptional signatures after a single prolonged social defeat stress exposure. Our findings were validated using cell-type specific approaches, such as RNAscope and qPCR from selected hippocampal regions and cell populations.

To the best of our knowledge, the dataset generated here represents one of the largest and most comprehensive resources of the adult Hipp, under baseline and stress conditions. In addition, it presents both wild-type and genetically modified knockout mouse lines for GR and MR, thus providing further possibilities of investigating the response to stress under these genetic modifications. The curated dataset is readily available on CELLxGENE [16], an interactive tool which allows users to easily download the data for further analysis, and to explore gene expression patterns across conditions and genotype. Our data can be accessed, visualized and downloaded here:

https://cellxgene.cziscience.com/collections/d245b35b-3cc1-4f47-aed6-68dfdadebb5f.

We envision that our data will accelerate research in the field of stress neurobiology and offer a unique opportunity for researchers to validate their findings or generate novel hypotheses. Ultimately, our resource and findings provide new insights into the molecular mechanisms underlying a healthy or abnormal response of the brain to an acute stressor, which can lead to more accurate, and reliable, molecular signatures to monitor disease progression.

## Results

### Generating a molecular catalog of the posterior hippocampus for stress research using scRNA-seq

**Fig. 1:**
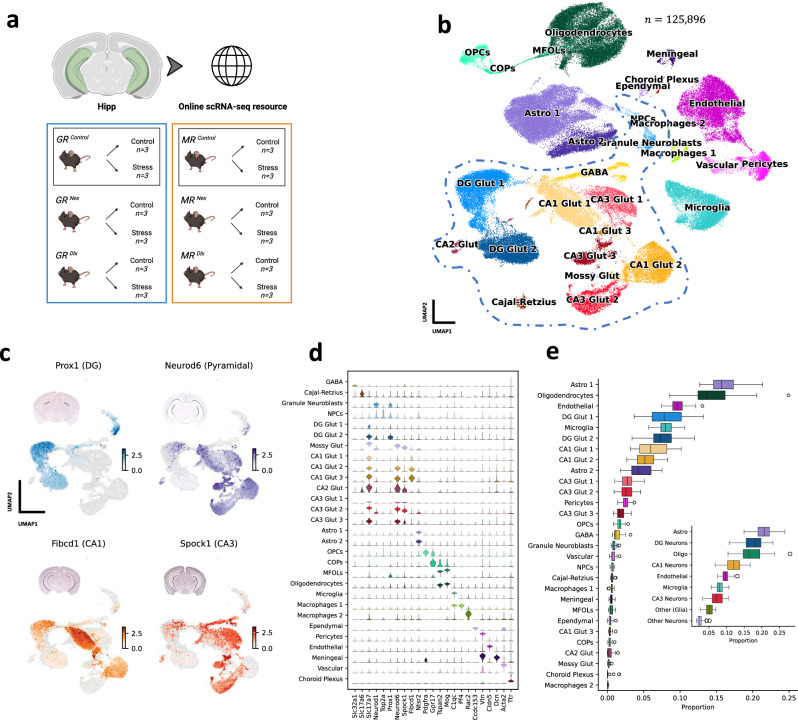
*Single-cell characterization of the adult murine hippocampus.* **a** Experimental design: A total of 36 mice (n = 6 per 6 genotypes) with a conditional GR and MR KO, in either glutamatergic (Nex^Cre^) or GABAergic (Dlx^Cre^) populations were used. GR^flox/flox^ and MR^flox/flox^ littermates were used as wild type controls (GR^Control^, MR^Control^). Triplicates were used for each condition (controls: n = 3; stressed: n = 3). **b** UMAP representation of the single-cell data collected from the adult posterior hippocampus. Cell type annotations are displayed on the data. Neuronal cells are grouped together by a blue dashed line. **c** Expression of the genes used to mark anatomical location within the adult hippocampus. The UMAPs show only the neuronal populations in the data. In-situ RNA hybridization profiles from the Allen Brain Atlas are shown on top of each panel. Cornu ammonis (CA) neurons are characterized by *Prox1* mRNA expression, while *Neurod6* marks neurons from the dentate gyrus (DG). CA neurons were further characterized in CA1 and CA3 neurons by leveraging the gene expression patterns of *Fibcd1* and *Spock1*. **d** Stacked violin plots showing the gene expression of various cell type markers in the annotated clusters. **e** Boxplot showing cell type proportions for each identified cell type across samples. The smaller (embedded) plot shows proportions after grouping various cell types in a coarse level of annotation.

With the aim of exploring the molecular and cellular identities of the posterior hippocampus and generating a unique resource for researchers interested in investigating the unknown molecular mechanisms underlying the response to a single prolonged social defeat stressor, we collected cell-type specific gene expression data from the Hipp of adult mice using scRNA-seq. We specifically designed this experiment to have multiple levels of complexity to address relevant open questions in the field of stress research, including: [1] the molecular and cellular landscape of the Hipp of adult mice, [2] cell-type specific transcriptional changes associated with acute stress exposure in wild-type mice, [3] the role of GR and MR in the acute stress response, and [4] the contribution of GR and MR to the stress-induced transcriptome in glutamatergic (excitatory) or GABAergic (inhibitory) neurons. To address these questions, we generated mice mutants lacking GR or MR, specifically in forebrain glutamatergic or GABAergic neurons. These were obtained by breeding *GR*^*flox/flox*^ *and MR*^*flox/flox*^ with *Nex-Cre* and *Dlx5/6-Cre* animals, respectively. In all experiments, the respective *GR*^*flox/flox*^ *and MR*^*flox/flox*^ littermates were used as wild type (WT) controls (Fig. 1a). For all single-cell experiments, triplicates were used for both control and stress conditions.

In total we collected single-cell data from 36 individual mice (Fig. 1a). After quality control and processing (*see Preprocessing and quality control*), two samples were removed, leaving us with a total of 34 samples and a dataset comprising 125,896 cells. The data was processed using the SCANPY [17] software, following established best practices [18], which included scran-normalization [19], log(x + 1) transformation, highly variable genes selection and batch correction. In order to gain more analytical power by leveraging higher numbers of cells for a thorough characterization of the adult Hipp, we opted to visualize, cluster and annotate the whole dataset at once. This analysis revealed distinct clusters of neuronal, glial and vascular cells (Fig. 1b). We further subdivided some of these clusters by analyzing their underlying substructure and gene expression patterns. For instance, we identified multiple clusters for glutamatergic neurons that were characterized by distinct marker gene expression profiles (e.g., CA1 Glut 1, 2 and 3). Furthermore, by leveraging the gene expression patterns of known anatomical marker genes of the hippocampus [20], we were able to further characterize various neuronal cell clusters by assigning them a specific anatomical location within the hippocampus. We used *Prox1* and *Neurod6* as markers of dentate gyrus (DG) and pyramidal (CA) neurons, respectively. We then further characterized pyramidal neurons into CA1 and CA3 neurons by investigating the expression patterns of *Fibcd1* and *Spock1* (Fig. 1c**)**. This anatomical characterization provided crucial context for targeted and accurate validation studies and offers an additional layer of characterization of neuronal cells. At the conclusion of this iterative process, a total of 29 clusters were obtained, within 3 major cell-type groups: neurons (GABA, Cajal-Retzius, granule neuroblasts, neural progenitor cells (NPCs), DG Glut 1-2, mossy Glut, CA1 Glut 1-3, CA2 Glut, CA3 Glut 1-3), glia (Astro 1-2, oligodendrocyte progenitor cells (OPCs), committed oligodendrocyte progenitors (COPs), myelin-forming oligodendrocytes (MFOLs), oligodendrocytes, microglia, macrophages 1-2), and vascular cells (ependymal, pericytes, endothelial, meningeal, vascular, choroid plexus). We further computed marker genes that are robust across genotypes and samples for each cell cluster (Supplementary Figure 1, Fig. 1d). Cell-type composition was consistent across samples (Fig. 1e, Supplementary Figure 2a), meaning that all cell clusters were well-balanced and represented across replicate samples (triplicates). After grouping similar clusters together to obtain a coarse annotation, we found that neurons represented the largest population of brain cells in the Hipp (39.72%), with DG Glut (17.59%), CA1 Glut (12.12%), CA3 Glut (7.37%), GABA (1.46%) and other neurons (1.17%). This was followed by astrocytes (20.97%), oligodendrocytes (16.59%), endothelial (9.61%), and microglia (8.11%).

After obtaining this high-resolution annotation, we observed several continuous phenotypes in the data that could be associated with differentiation trajectories, including oligodendrocyte maturation from OPCs. To explore the relationships between cell identities, we used the PAGA algorithm [21] to obtain a graph representation of the data which revealed connections between clusters (Supplementary Figure 2b). We found connections between clusters of vascular cells (pericytes, vascular, endothelial), neurons and macrophages. The strongest connections were found between cells in the oligodendrocyte trajectory (OPCs -> COPs -> MFOLs -> Oligodendrocytes). When we performed the same analysis exclusively on neuronal clusters, we observed numerous connections between neuronal types and a strong link between NPCs and granule neuroblasts (Supplementary Figure 2c).

### scRNA-seq reveals *Nrgn* as a marker of stress-reactive neurons in WT animals

**Fig. 2:**
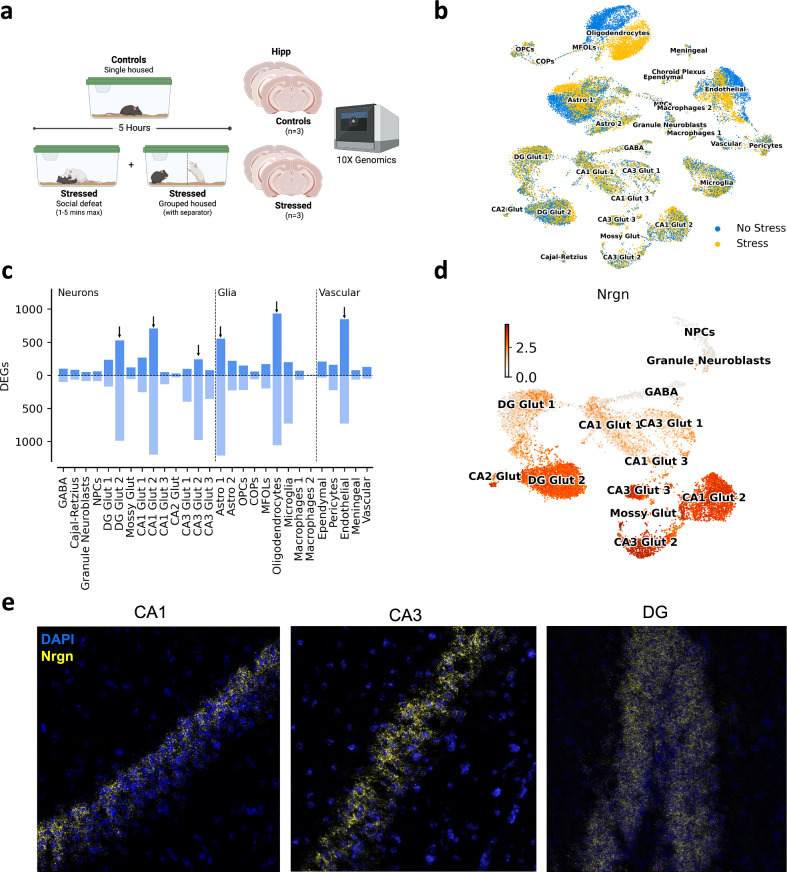
*Molecular characterization of the acute response to social stress.* **a** Diagram depicting the experimental protocol followed to obtain the data. **b** UMAP of the data with color representing the stress (yellow) and control (blue) conditions. Name of the clusters is overlaid in text. **c** Differential expression analysis of WT data between control and stressed animals. Cell types are grouped based on 3 major types (neurons, glia, and vascular). The bars pointing upwards show the number of upregulated genes, while those pointing downwards show the number of downregulated genes. The six clusters with the largest changes are marked with arrows. **d** UMAP of *Nrgn* expression in neurons. **e** Representative images of RNAscope validation: *Nrgn* (yellow) and DAPI (blue) mRNA expression in CA1, CA3 and DG neurons of the posterior hippocampus from an independent validation cohort (controls: n = 3; stressed: n = 3).

To study the transcriptional response to an acute stressor, we leveraged the data collected from all WT mice (n = 12), which included both GR^control^ (n = 6) and MR^control^ (n = 6) littermates (Fig. 1a). These mice were subjected to a single prolonged social defeat stress session. Following a single social defeat by a larger and territorial CD1 mouse, stressed mice (n = 6) were kept in the same cage as the resident CD1, but separated by a transparent perforated physical barrier, for a total of 5 h. Control mice (n = 6) were placed in a new cage and single housed for a matching period (Fig. 2a). As shown by individual plasma CORT levels, control animals habituate to the new environment, while defeated mice remined stressed due to a prolonged stressful situation (sensory and emotional), since the intruder mouse could still smell, hear, and see the aggressor resident CD1, throughout the 5 h prior to tissue collection (Supplementary Figure 2d). The brains of all mice (stressed and controls) were dissected for molecular characterization 5 h after social defeat stress or exposure a control environment. The 5-hour time point was specifically selected to capture the second wave of transcription initiated by immediate early genes (IEGs) [22], following a single prolonged social defeat stressor. This timepoint was selected to capture the second wave of transcriptional responses following stress, while also minimizing potential confounding activation of immediate early genes (IEGs) that can occur during tissue dissociation in single-cell RNA sequencing protocols. For our single cell sequencing experiments, the posterior hippocampus of stressed and control (non-stressed) mice was dissected and immediately processed. The single cell data showed its most significant changes in expression between control and stress in three populations: oligodendrocytes, astrocytes and endothelial cells (Fig. 2b). In order to quantify these effects, we performed a differential expression test between control and stressed animals in each annotated cluster. We modelled the data using a negative binomial generalized linear model with *diffxpy* [23] (*see Methods*) and performed a Wald test (Fig. 2c). This analysis confirmed that oligodendrocytes, astrocytes, and endothelial cells displayed the strongest differential expression signal among non-neuronal clusters. In neurons, an interesting pattern emerged, as we observed that certain subpopulations within glutamatergic neurons from the same type and anatomical location, responded to stress with a higher number of differentially expressed genes (DEGs). Specifically, CA1 Glut 2 (1911), CA3 Glut 2 (1219) and DG Glut 2 (1517) exhibited a substantially larger number of DEGs than similar subpopulations CA1 Glut 1 (525), CA3 Glut 1 (498) and DG Glut 1 (409) (Fig. 2c). However, due to power limitations in clusters with small numbers of cells, it is possible that our gene expression models in these clusters include larger levels of uncertainty and so fewer DEGs were recovered, as is the case for the small clusters in our dataset.

Characterizing these neuronal subpopulations further, we looked for markers that would explain the difference between them and the rest of neurons in their corresponding glutamatergic populations. This was done by ranking genes using a Welch’s t-test exclusively in neuronal cells. Interestingly, we found neurogranin (*Nrgn*) to be consistently and highly expressed across all three highly responsive glutamatergic neuron clusters (Fig. 2d), making it a prominent co-expressed gene within these stress-reactive populations. *Nrgn* codes for the neurogranin protein, which is expressed particularly in glutamatergic neurons and especially in dendritic spines [24]. This protein is involved in the protein kinase C pathway, as well as calcium and calmodulin signaling between neurons [25]. While its clinical relevance is not fully understood yet, it has been previously implicated in neurodegenerative disorders such as Alzheimer’s disease [26] and psychiatric disorders such as schizophrenia [27] and major depression [28]. To validate our findings, we used RNAscope to explore, visualize and quantify the mRNA expression of *Nrgn* in neurons from the three major areas of the hippocampus (CA1, CA3 and DG), in an independent cohort of WT mice. Our results show that *Nrgn* is expressed in some but not all neurons in the Hipp (Fig. 2e), with some neurons showing high and others low expression patterns. These results are consistent with our single cell analysis and suggest that the subpopulations with high *Nrgn* expression are the stress-responsive cells annotated as CA1 Glut 2, CA3 Glut 2 and DG Glut 2.

To further characterize the differences between the transcriptional response of high and low *Nrgn* neurons, we performed an enrichment analysis (see *Methods*) on the sets of DEGs in pairs of glutamatergic neuron subpopulations (e.g., DG Glut 1 vs. DG Glut 2, etc.) (Supplementary figures 3a-c). In CA3 neurons, we found that only DEGs from high *Nrgn* (CA3 Glut 2) neurons were significantly enriched for terms related to the organization and regulation of synaptic activity, behavior, cytoskeleton organization, and protein localization to the membrane. Terms that were enriched at a similar level in both clusters included processes related to brain development, mRNA processing and the regulation of translation. Similar analyses were performed for oligodendrocytes, astrocytes and endothelial cells (Supplementary Figure 4a-c).

**Fig. 3:**
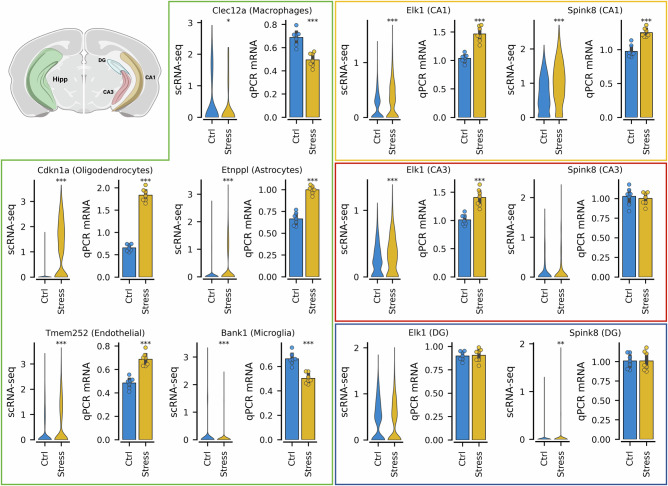
*Validation of differentially expressed genes in neuronal and non-neuronal cell types.* qPCR validation of selected differentially expressed genes after social stress exposure in neurons, glia and vascular cells from the posterior hippocampus, using an independent validation cohort (controls: n = 7; stressed: n = 7). For each gene a violin plot of gene expression in control and stress conditions in the single cell data is displayed on the left. On the right a barplot shows the qPCR results obtained to validate the data. Asterisks are used to denote the significance obtained using a two-tailed t-test. The green box (left side) contains genes validated in non-neuronal cells, from top to bottom and left to right: *Clec12a* (Macrophages, q = 0.02, Log2FC = -0.84), *Cdkn1a* (Oligodendrocytes, q = 0.00, Log2FC = 5.93), *Etnppl* (Astrocytes, q = 0.00, Log2FC = 1.53), *Tmem252* (Endothelial, q = 0.00, Log2FC = 1.16), *Bank1* (Microglia, q = 0.00, Log2FC = -0.85. The yellow, red and blue boxes (right side) contain genes validated in neuronal cells, from top to bottom and left to right: *Elk1* (CA1, q = 0.00, Log2FC = 0.59), *Spink8* (CA1, q = 0.00, Log2FC = 0.55), *Elk1* (CA3, q = 0.00, Log2FC = 0.24), *Spink8* (CA3, q = 0.32, Log2FC = 0.24), *Elk1* (DG, q = 0.11, Log2FC = -0.07), *Spink8* (DG, q = 0.00, Log2FC = 0.62). Log2FC: log2 fold change.

Finally, to reinforce the reliability of our single cell analysis, we selected a small group of DEGs for validation using qPCR in a third and independent cohort of 14 wild-type mice (controls: n = 7; stressed: n = 7). Since qPCR only allows for validation of gene expression in mixed populations of cells (bulk level), we chose genes in neuronal and non-neuronal populations that exhibited cell-type or anatomical region-specific expression and had large effect sizes. Following this approach, for non-neuronal cells we selected: *Cdkn1a* (oligodendrocytes), *Etnppl* (astrocytes), *Tmem252* (endothelial cells), *Bank1 (*microglia) and *Clec12a* (macrophages). In addition, since non-neuronal cells lack spatial localization across the hippocampus and are spread homogeneously, the tissue used for qPCR validation was collected from the whole Hipp. All six tested genes showed consistent directions of change with the results of our single-cell analysis (Fig. 3).

We performed a similar selection of genes to validate in neurons, but additionally focused on genes whose expression was absent in non-neuronal populations (glia and vascular cells), and with a subpopulation-specific stress response signal. That is, neuronal DEGs that were only significant in a particular anatomical location of the Hipp (DG, CA1 or CA3). To validate these genes expression patters, the tissue used for qPCR validation was collected specifically from the DG, CA1 or CA3 separately, offering us a degree of anatomical (region) and cell-type (neurons vs non-neurons) specificity (Fig. 3). Specifically, we selected *Elk1* which was differentially expressed in CA1 and CA3 (pyramidal neurons) but not DG, and *Spink8*, significant in CA1 and DG but not CA3. While *Elk1* was indeed significantly upregulated only in pyramidal neurons but not in the DG, *Spink8* exhibited a consistent upregulation in CA1 but not CA3 neurons or in the DG. Overall, the different levels of validation of our single-cell data (RNAscope and qPCR) significantly increase the confidence in the results of our scRNA-seq analyses, which we expect to become a useful resource for researchers working on the stress response in the hippocampus.

### Acute social stress exposure leads to *Sgk1* upregulation in mature oligodendrocytes

**Fig. 4:**
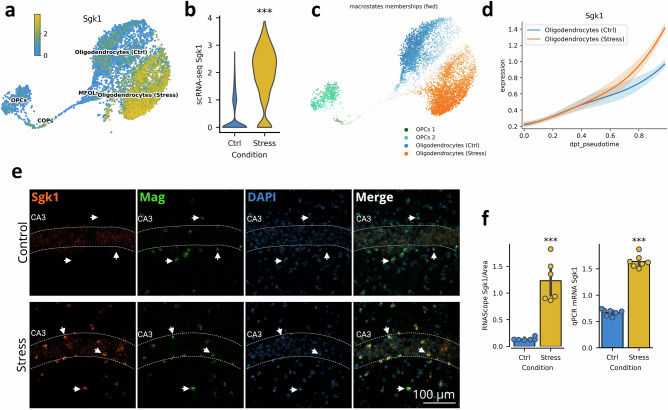
*Sgk1 is a molecular marker of the response to stress in oligodendrocytes.* **a** UMAP showing the expression of *Sgk1* mRNA in oligodendrocyte populations (OPC, COPs, MFOLs and mature oligodendrocytes) in WT mice. **b** Violin plot of *Sgk1* expression in mature oligodendrocytes in WT animals by condition. **c** UMAP showing transcriptional state probabilities for each cell as computed by CellRank. Two groups of mature oligodendrocytes (stress and control) as endpoints of the state trajectory are identified. **d** Gene expression trends of *Sgk1* along the pseudotime coordinate. The *Sgk1* gene is the top lineage driver of stressed oligodendrocytes. **e** Representative images obtained with RNAscope comparing *Sgk1* mRNA expression in control and stressed animals. *Sgk1* is shown in orange, DAPI (blue) is used as a marker for cell nuclei, *Mag* (green) is a marker of mature oligodendrocytes. **f** Quantification of RNAscope validation of *Sgk1* upregulation induced by acute stress.

Since the largest changes at the cell population level were observed in oligodendrocytes (Fig. 2b, c), we further analyzed the gene expression changes associated with this shift. This analysis revealed that serum and glucocorticoid-regulated kinase 1 (*Sgk1*) showed a strong association with the stress effect. Specifically, while *Sgk1* was expressed at relatively low levels across all four oligodendrocyte populations (OPCs, COPs, MFOLs, mature oligodendrocytes) under baseline conditions (non-stressed controls) (Fig. 4a), exposure to a single prolonged social defeat situation led to the significant elevation of *Sgk1* expression in mature oligodendrocytes (Log2FC = 2.44) (Fig. 4b).

To put this signal into the context of oligodendrocyte maturation, we performed a trajectory analysis on all cells in the oligodendrocyte clusters (OPCs, COPs, MFOLs and mature oligodendrocytes). To do so, we first computed the diffusion components on the cells in the oligodendrocyte trajectory and we found cells to split along the stress condition in the second diffusion component (Supplementary Figure 5a, b). We then used these components to compute the diffusion pseudotime [29], which covaried smoothly with the biological trajectory stemming from OPCs and ending with mature oligodendrocytes. Lastly, we computed transcriptomic states and probabilities using CellRank [30] on pseudotime. Highlighting the distinct control and stressed cellular states, the algorithm identified two end points in mature oligodendrocytes: one for oligodendrocytes from control mice, and a second one for oligodendrocytes from stressed animals (Fig. 4c). Inference of the main gene drivers behind each transcriptomic state revealed *Sgk1* to be a prominent stress-responsive gene in mature oligodendrocytes, which is consistent with the expression patterns of *Sgk1* across oligodendrocyte clusters. Our findings also imply that *Sgk1* is the first gene to be upregulated along the trajectory from OPCs to stressed mature oligodendrocytes (Fig. 4d) suggesting an early role in this process. For comparison, a similar analysis on *Cdkn1a*, another gene upregulated upon stress in oligodendrocytes (Log2FC = 5.93), reveals a delayed increase in expression compared to *Sgk1* in the pseudotime trajectory (Supplementary Figure 5c). To further validate these findings, we performed a quantification of *Sgk1* mRNA, specifically in oligodendrocytes from the Hipp of control and stressed mice, using RNAscope. In addition to *Sgk1*, we also stained for *Mag*, a well-known and validated marker of mature oligodendrocytes [31], and DAPI as a nuclei marker. Our results show relatively low levels of mRNA expression of *Sgk1* in a small group of mature oligodendrocytes and CA3 pyramidal neurons in the Hipp of control mice (non-stressed) (Fig. 4e). However, once mice were exposed to a single prolonged social defeat stress response session, the expression of *Sgk1*, specifically in mature oligodendrocytes, was significantly increased by a factor of 9.5 (Fig. 4f). These results, together with the upregulation of *Cdkn1a*, were further validated using qPCR (Fig. 3). Our results indicate that *Sgk1* is a molecular marker, in oligodendrocytes, for the effects of acute stress exposure and suggest that it may be a molecular driver for the response to social stressors in the Hipp of mice.

### Cell-type specific KOs of GR or MR lead to heterogeneous transcriptomic signatures in neurons after social stress exposure

After characterizing the cellular landscape of the Hipp and the molecular response to a single prolonged social defeat stressor in WT animals, we analyzed the molecular changes induced by cell-type specific GR or MR KO in control and stressed mice. First, we confirmed that the independent KOs worked as expected by assessing the expression of *Nr3c1* (GR gene) and *Nr3c2 (*MR gene*)* by in-situ hybridization (ISH) and by exploring the expression of the same genes at the single-cell level across all clusters and mouse lines (Ctrl, Nex, Dlx) (Supplementary Figure 6a-d). We observed that *Nr3c1* was expressed in WT animals, with varying intensity, across both neuronal and non-neuronal populations, and that its expression after GR KO in glutamatergic (GR^Nex^) or GABAergic (GR^Dlx^) neurons was blunted as expected (Supplementary Figure 6c). We observed similar patterns for *Nr3c2* in animals with an MR KO, with the main difference being that this gene was expressed mostly in neuronal cells (Supplementary Figure 6d).

**Fig. 5:**
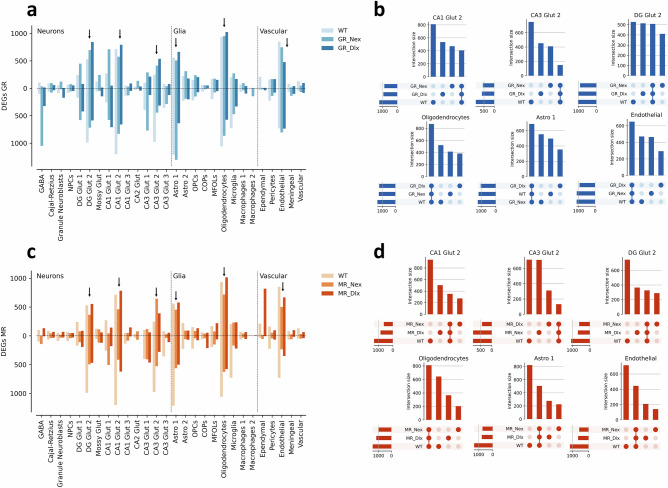
*The effect of stress after GR and MR knockouts.* **a** Bar plots showing the number of up and downregulated DEGs induced by stress in WT, GR-Nex and GR-Dlx animals. Black arrows highlight the six clusters with the highest number of DEGs (DG Glut 2, CA1 Glut 2, CA3 Glut 2, Astro 1, Oligodendrocytes and Endothelial cells). **b** Upset plots showing the sets of unique genes for each condition (WT, GR-Nex, GR-Dlx), plus the intersection set over all of them in the six selected clusters. **c** Bar plot showing the number of up and downregulated DEGs induced by stress in WT, MR-Nex and MR-Dlx animals. Black arrows highlight the six clusters with the highest number of DEGs. **d** Upset plots showing the sets of unique genes for each condition (WT, MR-Nex, MR-Dlx) plus the intersection over all of them in six selected clusters. DEG: differentially expressed gene.

To compare the effects of the receptor KO on stress response we compared the results of differential expression tests between stress and control animals within GR^Nex^, GR^Dlx^, MR^Nex^ and MR^Dlx^ KO lines (Supplementary Figure 6e) with those obtained from WT data for GR and MR separately. The total number of DEGs obtained in the GR^Nex^ KO and GR^Dlx^ KO mice show a similar trend as our previous results in stress-exposed WT mice. Specifically, the same six clusters (CA1 Glut 2, CA3 Glut 2, DG Glut 2, Oligodendrocytes, Astrocytes 1 and Endothelial) display the largest number of DEGs, indicating that the main responding subpopulations are consistent also under GR KO. Notable differences to the WT result include a substantial increase in the number of down-regulated genes in GABA cells in GR^Nex^ KO mice, and an increase of DEGs in CA1 Glut 1 (Fig. 5a). We decided to follow up the results in these six clusters and, to understand how heterogeneous the DEGs between these conditions were, we looked at upset plots of the unique sets of genes for WT, GR^Nex^ and GR^Dlx^, plus the intersection set over these three (Fig. 5b). We observed that the unique sets of genes were the biggest ones for neuronal clusters (CA1 Glut 2, CA3 Glut 2, DG Glut 2), while the intersection set was the largest one in non-neuronal clusters (Oligodendrocytes, Astrocytes and Endothelial). These results suggest that the KO of GR induced heterogeneous responses in neurons, while the response in non-neuronal clusters remained more similar to that in WT animals.

The same analyses for MR^Nex^ (KO in Glutamatergic neurons), MR^Dlx^ (KO in GABAergic neurons), and WT data showed similar patterns across the three mouse lines. Notable differences included an increase in up-regulated genes in ependymal cells in MR^Dlx^ animals and a consistent reduction in the number of down-regulated DEGs in the six most responsive clusters in both MR^Dlx^ and MR^Dlx^ specimens (Fig. 5c). Analysis of the uniqueness of the DEG set per cell type showed similar patterns as those observed in GR KO data, with the most heterogeneous responses occurring in neurons (Fig. 5d). In non-neuronal clusters, either the set of common genes or the WT set were the biggest ones, reflecting both the homogeneity of the responses across the KO conditions, and the reduction in DEGs compared to WT.

Furthermore, following up on one of the main results from our analysis on WT data, we investigated the effect of stress on *Sgk1* expression in oligodendrocytes. We observed that *Sgk1* displayed consistent up-regulation across all five conditions, suggesting that it is not affected by any of the KO conditions. Indeed, *Sgk1* expression patterns in control and stress animals across the five conditions showed that the up-regulation pattern is consistent across KOs (Supplementary Figure 6f). However, *Sgk1* expression in control GR^Nex^ animals was slightly elevated compared to WT, suggesting a baseline effect after GR KO in glutamatergic neurons.

### GR and MR target genes display expression changes in the response to acute stress after cell-type specific KO

**Fig. 6:**
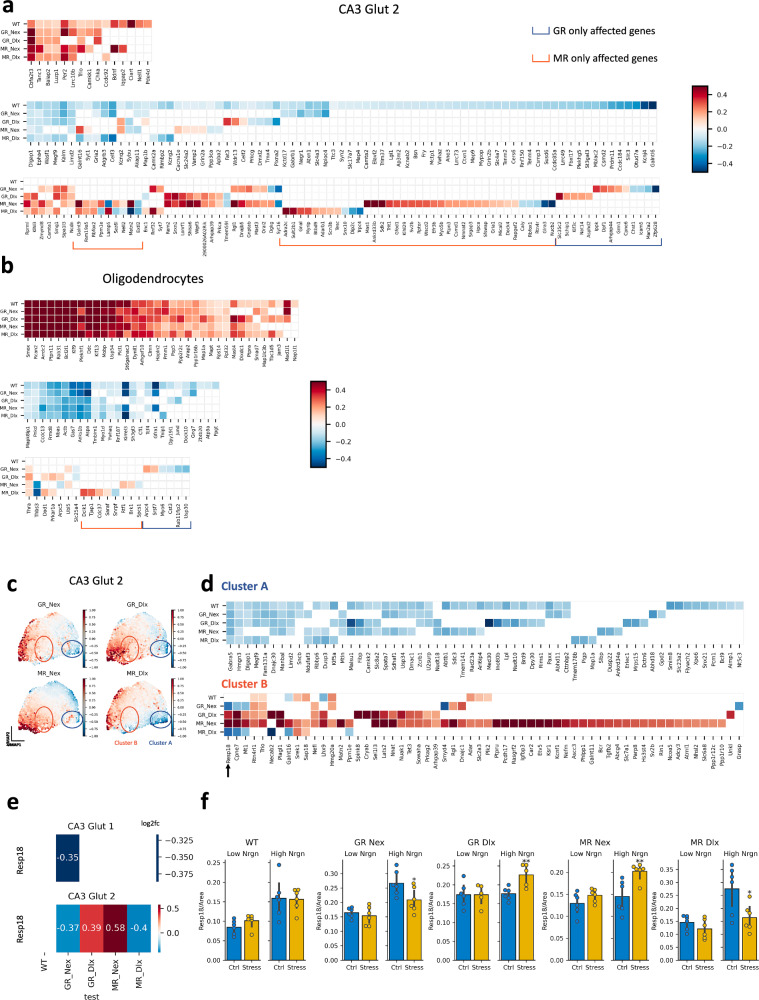
Expression patterns of GR and MR targets across KO conditions. (**a**-**b**) Hypotheses-driven analyses of validated GR and MR targets. Heatmaps show the Log2FC of validated GR and MR targets (x-axis) in each tested condition (y-axis) in CA3 Glut 2 neurons and oligodendrocytes. Log2FC is only shown for DEGs, in at least one of the five conditions. Genes are separated between three groups: (top) genes that were upregulated in WT, (middle) genes that were downregulated in WT, (bottom) genes that were not significant in WT. With brackets we highlight genes whose expression is affected by either only GR (blue) or only MR knockouts (red). (**c**-**d**) Data-driven exploration of differential expression patterns across KO conditions. (**c**) UMAP of genes that were DE in at least one of the five conditions in CA3 Glut 2 neurons. The features analyzed are the Log2FC values in each condition. The Log2FC values in GR-Nex, GR-Dlx, MR-Nex and MR-Dlx conditions are shown in color. From a cluster analysis two clusters, A (blue) and B (red) are singled out. **d** Heatmap showing Log2FC patterns across conditions of genes in clusters A and B. *Resp18* was selected for further validation. **e** Log2FC of *Resp18* across the five tested conditions in CA3 Glut 1 and CA3 Glut 2 neurons, with numeric values. **f** RNAscope validation of *Resp18* differential expression in CA3 Glut 2 neurons of the posterior hippocampus using an independent validation cohort of 30 mice (n = 6 for 5 genotypes) with triplicates per condition (controls: n = 3; stressed: n = 3). We use *Nrgn* expression to differentiate between CA3 Glut 1 (low *Nrgn*) and CA3 Glut 2 (high *Nrgn*) neurons in RNAscope readings. DEG: differentially expressed gene. Log2FC: log2 fold change.

To further explore the complex results obtained with our five-way differential expression test setup, we performed a hypothesis-driven analysis whereby we investigated the patterns of expression in response to stress of known GR and MR target genes across genotypes. We obtained a list of GR and MR target genes generated from a ChIP-Seq study of the rodent hippocampus [32, 33]. We grouped these genes according to their cell-type-specific expression patterns and then identified, for each cluster, a subset of genes that were up-regulated in WT, a second subset of genes down-regulated in WT mice, and a subset of genes that were not differentially expressed in WT (see *Methods*). In CA3 Glut 2 neurons this analysis identified heterogeneous patterns of GR and MR target genes across the five mouse lines (Fig. 6a), especially in the subset of genes that were not significant in WT but significant in at least one of the GR or MR KO lines. A substantial amount of these genes appears to be affected exclusively by one of the two receptor (GR/MR) KOs. Similar results were obtained for the other neuronal clusters CA1 Glut 2 and DG Glut 2 (Supplementary Figure 7). In contrast, the same analysis for oligodendrocytes (Fig. 6b**)** revealed a more homogeneous response across the five lines. Other non-neuronal clusters such as astrocytes and endothelial cells displayed similar patterns albeit with a reduced presence of GR and MR targets, confirming that the KO affects transcriptomic patterns mainly in neurons (Supplementary Figure 8).

### Data driven analysis reveals genes with varied response to stress across KO lines

To expand our investigation of KO-line-specific gene expression changes beyond GR and MR targets, we expanded the analysis to all genes in a data-driven manner by embedding and clustering genes to identify both gene sets that behave similarly across conditions (e.g., Clust A in Fig. 6c, d), and gene sets that display heterogeneous patterns (e.g., Clust B in Fig. 6c, d). In CA3 Glut 2 neurons, this data-driven analysis identified several genes with cKO-specific stress responses that characterize the differential cell-type-specific effect of MR and GR KOs (Fig. 6c, d). As a proof-of-concept validation of this data-driven analysis, we selected *Resp18* for validation using RNAscope. This gene was significant in all four KO conditions while being differentially regulated in different directions after stress (Clust B) and displayed the largest effect sizes. Specifically, *Resp18* was up-regulated in CA3 Glut 2 after stress in GR^Dlx^ (q < 0.05, Log2FC = 0.39) and MR^Nex^ (q < 0.001, Log2FC = 0.58) animals, while it was down-regulated in GR^Nex^ (q < 0.01, Log2FC = -0.37) and MR^Dlx^ (q < 0.05, Log2FC = -0.40). This effect was specific to CA3 Glut 2, while *Resp18* was mostly not significantly affected by stress in CA3 Glut 1 neurons, except for down-regulation in GR^Nex^ (q < 0.001, Log2FC = -0.35) (Fig. 6e). As stated earlier, the defining characteristic of CA3 Glut 2 over CA3 Glut 1 cells is a high level of *Nrgn* mRNA expression. Using RNAscope, we were able to identify low *Nrgn* (CA3 Glut 1) and high *Nrgn* (CA3 Glut 2) neurons in CA3, which ultimately allowed us to obtain levels of *Resp18* mRNA expression in the two subpopulations of CA3 neurons. When we compared *Resp18* expression between control and stressed samples, we observed that the patterns obtained with RNAscope matched those found in the single-cell data in both low *Nrgn* (Glut1) and high *Nrgn* (Glut2) CA3 neurons. Notably, all five patterns across KO lines were successfully validated (Fig. 6f). Thus, our analysis reveals distinct signatures of MR and GR receptor KOs on the murine Hipp stress response. Our data-driven analysis represents a viable approach to distill information in a complicated experimental setup like the one used to generate the presented dataset. Importantly, the successful validation of a gene with complex and changing patterns of differential expression across the tested conditions, increases the confidence in the quality, processing, annotation and analysis of the dataset.

## Discussion

The response to stressful stimuli is crucial to the life of the organism and is a highly orchestrated physiological and behavioral process, which has been the subject of intense research for many years [34]. A dysfunctional stress response can impact the quality of life of the affected people and develop into diverse and severe psychiatric conditions. Despite tremendous effort in this direction, the underpinning complex molecular mechanisms of the central stress response remain to be fully elucidated, mainly due to their complexity [35]. In this study, we generated a comprehensive and high-quality single-cell resource for the field of stress research. This resource is available to the scientific community via an easy-to-use interactive browser tool, which can be used to explore the data in detail and will help with generating and testing new hypotheses. We gathered data from the Hipp of 34 adult mice and catalogued 125,896 cells, identifying 29 cell-types. By leveraging known anatomical markers, we were able to also assign neurons of the same type to different anatomical locations, which enabled targeted validation. The dataset we generated is, to the best of our knowledge, the largest single-cell characterization of the adult murine Hipp, an area strongly linked to the organism central stress response [15, 36, 37], and fundamental for studying the interplay between stress hormone receptors. Using a dedicated experimental design, we collected data across stress condition and GR or MR conditional KOs in either GABAergic or glutamatergic neurons. Following a bottom-up approach, we first investigated in detail the response to a single prolonged social defeat stressor in WT animals across cell identities, we then analyzed the changes induced by the KOs with a hypothesis-driven and a data-driven approach, with the goal of gaining a more holistic view.

An analysis of the acute stress response in WT animals identified three non-neuronal clusters, which had a large amount of DEGs induced by a single prolonged social defeat stressor: astrocytes, oligodendrocytes and endothelial cells. Interestingly, we also identified three subpopulations of glutamatergic neurons across CA1, CA3 and DG, which exhibited a stronger response to acute stressor compared to other neuronal cell types. After an investigation of these subpopulations, the gene *Nrgn* emerged as a common marker, with higher expression registered in all three groups compared to the rest of neurons. We validated these findings using RNAScope. *Nrgn* codes for the neurogranin protein and has been linked in previous studies to psychiatric and neurodegenerative disorders but had never been so clearly observed as a marker of stress-responding neurons. This is most likely due to the cell-type specific effects of *Nrgn* across neurons in the hippocampus, whose differential expression could be diluted or distorted in other studies using brain homogenates (bulk level). In the stress response of WT mice, we also observed a strong up-regulation of *Sgk1* induced by an acute stressor in oligodendrocytes. This was confirmed by both a differential expression analysis and a trajectory inference analysis, and appeared to be consistent across KO conditions. We validated this effect using both RNAScope and qPCR techniques. While *Sgk1* has been already shown to play a potential role in the cellular response to stressful stimuli and has been associated with stress-related psychiatric disorders [38–40], most studies have focused on its function in neuronal populations and have not investigated its role in oligodendrocytes [41, 42]. Furthermore, our findings also suggest that these cell-type-specific molecular changes could be diluted or masked in alternative studies using bulk tissue analyses.

In this study we set out to analyze how cell-type specific genetic manipulation of GR and MR receptors would impact the central response to a single prolonged social defeat stressor. These receptors are known as key players of this mechanism [43–46] and are thus subject of great attention from the scientific community. When analyzing the changes induced by GR or MR KOs, we first looked for large-scale differences in differential expression patterns across clusters. We observed that the six responding clusters identified in the WT analysis (CA1 Glut 2, CA3 Glut 2, DG Glut 2, Oligodendrocytes, Astrocytes 1 and Endothelial) still exhibited the largest responses in both GR and MR KO conditions. Importantly, while the lists of DEGs induced in the different conditions were fairly homogenous in non-neuronal clusters, we observed high degrees of heterogeneity in the responses of neurons. This led us to explore these cell-types further using two different approaches. Following a hypothesis-driven approach, we looked at the differential expression patterns across conditions of known GR and MR targets. We investigated the six clusters identified in the previous analyses and, similarly to the previous analysis, we found very heterogeneous responses in neurons where many genes appeared to be impacted by the KOs. Patterns that emerged from non-neuronal cell types were, on the other hand, more homogeneous, which is expected, since the KO was induced specifically in neurons. Considering that the stress-induced changes in gene expression patterns observed in glial and vascular cells seem relatively independent of MR and GR expression in glutamatergic or GABAergic neurons, it is likely that they are driven by GR signals in these populations, since MR is not highly expressed in these cells. It is also possible that these effects are caused by other stress hormones, such as corticotropin-releasing hormone or catecholamines, among others.

Going further, with a data-driven approach, we looked for interesting patterns across conditions without restricting ourselves to known targets of GR or MR. In order to further test our analysis, we focused on one neuronal subpopulation (CA3 Glut 2). We grouped together genes by their similarity in differential expression patterns across conditions using a cluster analysis. We thus identified groups of genes which exhibited complex patterns. As a proof-of-concept, we opted to validate the gene *Resp18* across the five conditions using RNAScope. This gene was not significantly differentially expressed in WT, but it displayed an interesting pattern of expression across KO lines, after acute stress exposure. More specifically, *Resp18* was found to have a strong and significant upregulation in GR^Nex^ and MR^Dlx^ deficient mice, while showing the opposite effect (down-regulation) in GR^Dlx^ and MR^Nex^ lines. Most importantly, our validation experiments confirmed that the effects of *Resp18* were only significant in the sub-population of stress responsive neurons of CA3 with high *Nrgn* mRNA expression. Our results not only confirm the findings from our computational analysis but highlight the presence of stress-responsive neurons in the Hipp. Interestingly, our data revealed a robust transcriptional response to stress in CA3 Glut 2 neurons, despite the relatively low expression levels of GR in this hippocampal subregion—a finding that may appear counterintuitive given the established literature and our own expression data. This observation suggests that GR abundance alone may not fully account for the magnitude of stress-induced transcriptional changes. Instead, it is possible that CA3 neurons exhibit heightened sensitivity to upstream signaling cascades or indirect GR-mediated effects, potentially through network-level interactions or paracrine mechanisms involving other cell types. Moreover, the identification of Nrgn as a marker of stress-responsive neurons and the successful validation of Resp18 expression changes in CA3 Glut 2 neurons across multiple GR and MR knockout lines further support the functional relevance of this subpopulation. These findings underscore the importance of considering both receptor expression and downstream transcriptional dynamics when interpreting stress responses in distinct neuronal populations. Regarding the analysis, we think that both the hypothesis- and data-driven approaches represent two valid ways to distill information from a complex and vast dataset like the one we generated. After exploring these large-scale changes, we narrowed down to a particular cluster and gene to validate as a proof-of-concept.

While our findings offer valuable insights into the cell-type-specific stress response, it is important to consider several limitations that may influence interpretation and future applications. First, our study focused on the posterior hippocampus, which includes both dorsal and ventral regions, but does not fully capture the ventral-most areas most strongly implicated in emotional regulation. Second, the stress paradigm involved a prolonged 5-hour exposure following a brief social defeat session. This design was intended to simulate a sustained stressful experience, both sensory and emotional, and to capture the second wave of transcriptional responses downstream of immediate early gene (IEG) activation. While this timing helps minimize confounding IEG induction during tissue dissociation, it may also reflect compensatory or secondary processes rather than primary stress responses. Third, although our single-cell RNA-seq dataset included 36 samples across multiple genotypes, each experimental group (stress vs. control) consisted of only three biological replicates. While this aligns with current best practices in single-cell transcriptomics, it limits statistical power and increases susceptibility to inter-individual variability. To mitigate this, we conducted RNAscope and qPCR validations in two additional and independent cohorts, which substantially strengthen the robustness of our findings. Fourth, due to the use of Nex-Cre lines, GR and MR deletions were not achieved in the dentate gyrus, a region that remained intact in mutant animals and may have contributed to residual receptor expression and transcriptional activity. Finally, all experiments were conducted in male mice, which limits the generalizability of our findings across sexes, particularly given known sex differences in stress responses and MR/GR signaling.

Our dataset represents the most comprehensive characterization of the mouse hippocampus under stress condition to date. We expect researchers in the field to leverage the data to generate hypotheses and validate them using our thoroughly validated scRNA-seq reference. While the results obtained in our analyses are novel, we believe that the vastness of the data contained in this dataset will be further explored by the stress researchers and will spark numerous opportunities for new studies and discoveries. Stress-related disorders remain a burden for millions of people worldwide, and we expect the current extensive resource to further accelerate current efforts made to unravel the mechanisms behind the stress response and contribute to the potential discovery of therapeutic solutions.

## Methods

### Animals and housing

All experiments were performed in accordance with the European Communities’ Council Directive 2010/63/EU. Animal experiments were approved by the Ethics Committee for the Care and Use of Laboratory Animals of the government of Upper Bavaria (Munich, Germany), or the Institutional Animal Care and Use Committee of The Weizmann Institute of Science (Rehovot, Israel). Male CD-1 (ICR) mice, aged between 7-10 weeks old were used for all experiments. Mice were group housed in IVC cages under specific-pathogen-free conditions. The room was temperature (23 ± 1 °C) and humidity-controlled (55 ± 10%) and on a 12-h light-dark cycle (lights on at 7:00). Food and water were provided ad libitum. Original cohort: Single-cell experiments (36 mice); n = 6 for 6 genotypes with triplicates per condition (controls: n = 3; stressed: n = 3) (Fig. 1a). Second cohort: RNA-Scope validation (30 mice); n = 6 for 5 genotypes with triplicates per condition (controls: n = 3; stressed: n = 3). Third cohort: qPCR validation (n = 14): wild-type mice (controls: n = 7; stressed: n = 7).

### Generation of GR and MR KO mice

Transgenic knockout mice were generated by breeding GR^flox/flox^ and MR^flox/flox^ with Dlx5/6-Cre and Nex-Cre animals, as previously described [47]. The Nex-Cre promoter is under the control of the *Neurod6* gene, which is not expressed in the adult dentate gyrus of the hippocampus. As there is no Cre expression in the dentate gyrus in GR^Nex^ and MR^Nex^ mouse lines, neither GR nor MR were deleted in this hippocampal subregion (**see** Supplemental Fig. 6a-d). In all experiments, the respective GR^flox/flox^ and MR^flox/flox^ littermates were used as wild type controls. For all single-cell experiments, triplicates were used for both control and stress conditions.

### Acute social stress experiment

To investigate the mechanisms behind a single prolonged social defeat stress response, male resident CD1 animals were trained for aggression. For five consecutive days a naïve male CD1 animal was placed in the home cage of the male resident CD1 animal for approximately 5 min to induce aggressive attacks. Groups of animals were randomly assigned to the single social defeat condition or the control condition. The animals to be acutely defeated were placed in the home cage of the resident aggressor animal, eliciting aggressive attacks towards the intruder animal. Following a single defeat by a larger and territorial CD1 mouse, stressed mice were kept in the same cage as the resident CD1, but separated by a physical barrier, for a total of 5 h. This resulted in a prolonged stressed situation (sensory and emotional), since the intruder mouse could still smell, hear and see the aggressor resident CD1. A single defeat session is defined as when the intruder animal displayed classical defeat posture. For all experiments, animals were defeated at 9 a.m. and tissue was collected five hours after the social defeat stress. Control mice were placed in a new cage and single housed for a matching period (5 h) (Fig. 2a).

### Plasma CORT measurements

Blood sampling was performed during end point (2:00 p.m.) by collecting blood from the heart of each mouse before perfusion with PBS. Blood samples were kept on ice and centrifuged at 4 °C. Plasma (10 μl) was removed for measurement of CORT. All plasma samples were stored at −20 °C until CORT measurement. Concentrations of CORT were quantified by radioimmunoassay (RIA) using a CORT double antibody 125I RIA kit (sensitivity: 25 ng/ml; MP Biomedicals Inc.) following the manufacturer’s instructions. Radioactivity of the pellet was measured with a gamma counter (Wizard2 2470 Automatic Gamma Counter; Perkin Elmer). All samples were measured in duplicate, and the intra- and interassay coefficients of variation were both below 10%. Final CORT levels were derived from the standard curve.

### Cell capture, library preparation and high-throughput sequencing

Generation of single-cell suspensions and cell capture was performed as previously described [11, 48–50]. Five hours after social defeat stress the mice received an isoflurane overdose and subsequently perfused with ice-cold PBS. The brains were removed and placed in ice-cold oxygenated artificial cerebral spinal fluid (aCSF). The extracted tissue from each animal was kept separately and was maintained in the same ice-cold aCSF solution throughout the entire dissection and dissociation procedure. Throughout the experiment, the aCSF was oxygenated with a 5% CO2 in O2 mixture. 1000 μm thick slices were cut using a VT1200/S Leica vibratome. The Hipp (-2.46 mm Bregma to -3.52 mm Bregma) was manually extracted under guidance of a stereo microscope (M205C, Leica). The tissue was dissociated with the Papain dissociation system (Worthington) for 35 min at 37 °C in a shaking water bath according to the provided protocol. Cells were filtered with 30 μm filters (Partec) and placed in cold aCSF. For sample loading, cell suspensions with ~1,000,000 cells/μl were used. Samples were loaded onto individual lanes of a 10X Genomics Chromium chip (10x Genomics), according to the manufacturer’s recommendations. To avoid batch effects, all samples were loaded and processes on the same chip. Reverse transcription and library preparation were performed according to the protocol provide with the 10X Genomics Single-Cell v2.0 kit (10x Genomics). Library concentrations and fragment length were determined by qPCR using KAPA Library Quant (Kapa Biosystems) and Bioanalyzer High Sensitivity DNA kit (Agilent), respectively. The libraries were pooled and sequenced on a single lane of an Illumina NovaSeq 6000 System generating 100–base pair paired-end reads.

### Preprocessing and quality control

We used CellRanger (v 2.0.2) with default parameters to preprocess the raw data. The mm10 (release 96) assembly and gene annotation used to align and quantify the data were provided by 10X Genomics. The analysis of the data was performed using Scanpy [17] (1.7.1), using the AnnData data structure (0.7.4). For quality control we explored the joint distributions of count depth, number of expressed genes and mitochondrial gene fraction in all samples of our dataset. We opted to tailor the cut-off thresholds for the filters for each sample independently. The chosen values for said thresholds can be found in the *Supplementary Material*.

Subsequently, we used Scrublet [51] (0.2.1) to detect and remove potential doublets in our data. The tool was run with the default parameters for each sample separately. After noticing that sample *GR_Nex_395* did not integrate well with the rest of the sample we opted to remove it from the dataset and further analyses. We normalized the data using the pooling-based normalization tool Scran [19] (1.18.5). To create the groups needed by the algorithm, we temporarily normalized the data using median count normalization and log(x + 1)-transformed it. We performed *Louvain* [52] clustering with a resolution of 0.5. The clusters thus obtained were then passed to Scran. We opted to normalize the whole dataset at once since the data did not display strong batch effects that could hinder or bias the *louvain* clustering step. We used the size factors returned by Scran to normalize the data which was subsequently log(x + 1)-transformed. The dataset was batch-corrected with Scanpy’s implementation of ComBat [53], using the KO key as batch (GR, MR), samples that fall under the same KO key were sequenced together and displayed no discernible batch effects. This linear batch correction method proved sufficient thanks to the good quality of the raw data and relatively mild batch effects between samples. Finally we selected 4000 highly variable genes using the *sc.pp.highly_variable_genes* of Scanpy, with flavor *cell_ranger*.

### Clustering, subclustering, and cluster annotation

We generated the final UMAP [54] visualization of our data with SCANPY using a kNN graph with 15 neighbors. We used the same graph to cluster the data using the python implementation of the Louvain algorithm. We opted to start with a resolution of 0.5. We detected the marker genes of each cluster by using Scanpy’s *rank_genes_groups* function with default parameters (Welch’s t-test). We annotated these clusters using a set of literature-based marker genes. Subsequently, we iteratively investigated each cluster by subclustering the data within it at a finer resolution. We then found the main differences between these subclusters by looking at the marker genes found by Scanpy’s *rank_genes_groups* function using only the rest of the cells in the cluster as a reference. With this process we were able to obtain a finer and more detailed annotation of our data. After this step we opted to merge similar subclusters under the same label.

By leveraging literature-derived markers of anatomical location within the hippocampus we were able to add spatial information to the clusters of neural cells found in our data. We used genes *Prox1* and *Neurod6* as markers of dentate gyrus (DG) neurons and pyramidal cells respectively. We then further characterized pyramidal cells in CA1 and CA3 neurons by looking at the expression patterns of *Fibcd1* and *Spock1*.

### Trajectory inference

We performed trajectory inference on the group of cell-types related to oligodendrocytes development: OPCs, COPs, MFOLs, Oligodendrocytes. We used CellRank [30] (1.2.0), a toolkit based on Markov state modelling of single-cell data. We used the PalantirKernel, which allowed us to perform the analysis using a pseudotime [29] covariate. To obtain pseudotime we use diffusion pseudotime (*sc.tl.dpt*) on a kNN graph of our subset of the data (k = 50).

### Differential expression testing

To quantify the effect of stress on gene expression we used Diffxpy [23] (0.7.4) for differential expression testing. We performed pairwise tests for each cluster between *Ctrl* and *Stress* cells within each set of perturbations (e.g., WT, GR^Nex^, etc.). We used a Wald test after fitting a negative binomial GLM to the count data with the following formulation:

$${Y}_{{ij}} \sim 1+{C}_{j}+{S}_{j}$$

Where Cj is the condition label (Ctrl or Stress) and Sj is the sample covariate of the cell. We constrained the model so that the difference between the variances from each sample equaled 0. We offset the GLM by factoring in the size factors obtained during the normalization step. To improve numerical stability, we centered these size factors so that their mean was equal to 1 before each test. For downstream visualizations and analyses we filtered out genes with an absolute Log2FC < 0.1 and with mean expression < 0.1 in both control and stress data.

### Hypothesis-driven analysis of GR and MR KO DEGs

We used a list of GR and MR genes obtained from a study conducted on rat male brains which aimed to identify GR and MR targets using CHiP-Seq [32]. We joined the lists of peaks obtained from BLAM (baseline morning) and BLPM (baseline afternoon) conditions and subset the genes to those present in the dataset. From this joint list we grouped genes by their expression patterns, looking for neuron-specific genes, oligodendrocytes-specific genes and so on. We did so by obtaining a matrix of mean gene expression in each cluster for each gene and performing a gene-wise cluster analysis. For visualization, we separated genes in three groups: genes that were up-regulated in WT, genes that were down-regulated in WT and genes that were not significantly differentially expressed in WT. We simplified the analysis by taking only genes that were significant in at least one of the 5 tested conditions. For this we used an unfiltered list of DEGs (no Log2FC or mean expression filter). In the visualized heatmaps we ranked genes within each of these groups by the number of conditions in which they were significant and by their Log2FC.

### Data-driven analysis of KO DEGs

In our data-driven analysis we obtained, for each cluster, a matrix of genes by tested condition with the Log2FC obtained from the differential expression tests as features. We then performed a cluster analysis on this matrix, which grouped clusters by their patterns of differential expression across conditions. We thus identified genes with homogenous responses (e.g., up-regulated in all five conditions) and also genes with more complex patterns (e.g., up-regulation in certain conditions with down-regulation in others).

### Gene ontology and pathway enrichment

We used Metascape [55] to look for significantly enriched GO:bp [56] (biological processes) in our sets of genes that were differentially expressed after stress in each condition. In order to gain more statistical power, we used the full set of DEGs without any filtering step.

### RNAscope fluorescence in situ hybridization

Brains were removed and snap frozen using 2-methylbutane and stored at -80 °C until use. Frozen brains were cut with a cryostat in 20 µm thick cryosections. RNAscope fluorescence in situ hybridization was performed according to the manufacturer’s instructions supplied with the RNAscope Flourescent Multiplex Reagent Kit (Advanced Cell Diagnostics, Newark, Ca, USA), and described in previously [*71]*. The following detection probes were used; *Nrgn* (cat. no. 499441-C1), *Resp18* (cat. no. 493871-C3), *Slc17a7* (cat. no. 416631-C2), *Sgk1* (cat. no. 434791-C1), *Mag* (cat. no. 446451-C3). Images were acquired with a Zeiss confocal microscope using a 20x objective using identical settings for all samples. The signal intensity of a target gene (*Nrgn*, *Resp18* and *Sgk1*) was measured in individual cells positive for the appropriated cell marker gene (*Mag, Slc17a7*, or DAPI). Quantification of the images was done with Fiji. The mean signal intensity of all cells measured in an imaged field were averaged. For each condition, brain samples collected from three animals. Two cryosections were selected and individually stained, each of which images were taken bilaterally.

### Reverse transcription and quantitative real-time polymerase chain reaction

Messenger RNA (mRNA) samples were extracted using the miRNeasy kit according to the manufacturer’s instructions (Qiagen). Quantification of mRNA levels (bulk) was carried out using quantitative real-time PCR (qRT PCR). Total RNA was reverse transcribed using the High-Capacity cDNA Reverse Transcription Kit (Applied Biosystems). Real-time PCR reactions were run in triplicate using the ABI QuantStudio6 Flex Real-Time PCR System and data was collected using the QuantStudio Real-Time PCR software (Applied Biosystems). Expression levels were calculated using the standard curve, absolute quantification method. The geometric mean of the endogenous expressed genes Rpl13 and Gapdh were used to normalize the data.

### Quantification and statistical analysis

All graphs represent mean ± SEM. All graphs show individual data points throughout the paper. For each graph, the sample size and statistical test used are described in the corresponding figure legend. When ANOVA was used, Shapiro-Wilk normality test was used to verify normality in data distribution. In case normality was violated non-parametric test such as Kruskal-Wallis rank sum test was used. No method was used to predetermine whether the data met assumptions of the statistical approach.

## Supplementary information

Supplemental Material

## Data Availability

All data needed to evaluate our conclusions are present in the paper or the Supplemental Materials. Additional related data may be obtained from the lead contact upon request. Processed data can be accessed, visualized and downloaded here: https://cellxgene.cziscience.com/collections/d245b35b-3cc1-4f47-aed6-68dfdadebb5f. The code used for processing the data and generating our results, as well as all tables with DEGs and enrichment analysis results are made available here: The code: https://github.com/theislab/MRGR_stress_analysis. Tables: https://zenodo.org/records/15213233

## Code availability

All data needed to evaluate our conclusions are present in the paper or the Supplemental Materials. Additional related data may be obtained from the lead contact upon request. Processed data can be accessed, visualized and downloaded here: https://cellxgene.cziscience.com/collections/d245b35b-3cc1-4f47-aed6-68dfdadebb5f. The code used for processing the data and generating our results, as well as all tables with DEGs and enrichment analysis results are made available here: The code: https://github.com/theislab/MRGR_stress_analysis. Tables: https://zenodo.org/records/15213233.
