# Supplementary material for: Single-cell characterization of the adult male hippocampus suggests a prominent, and cell-type specific, role for Nrgn and Sgk1 in response to a social stressor
Source: Mol Psychiatry. 2026 Jan 19;31(3):1823–36. doi: 10.1038/s41380-025-03417-y (PMC12916315; doi:10.1038/s41380-025-03417-y)
Supplement: Supplementary file 1 — Supplemental Material [file 41380_2025_3417_MOESM1_ESM.pdf]

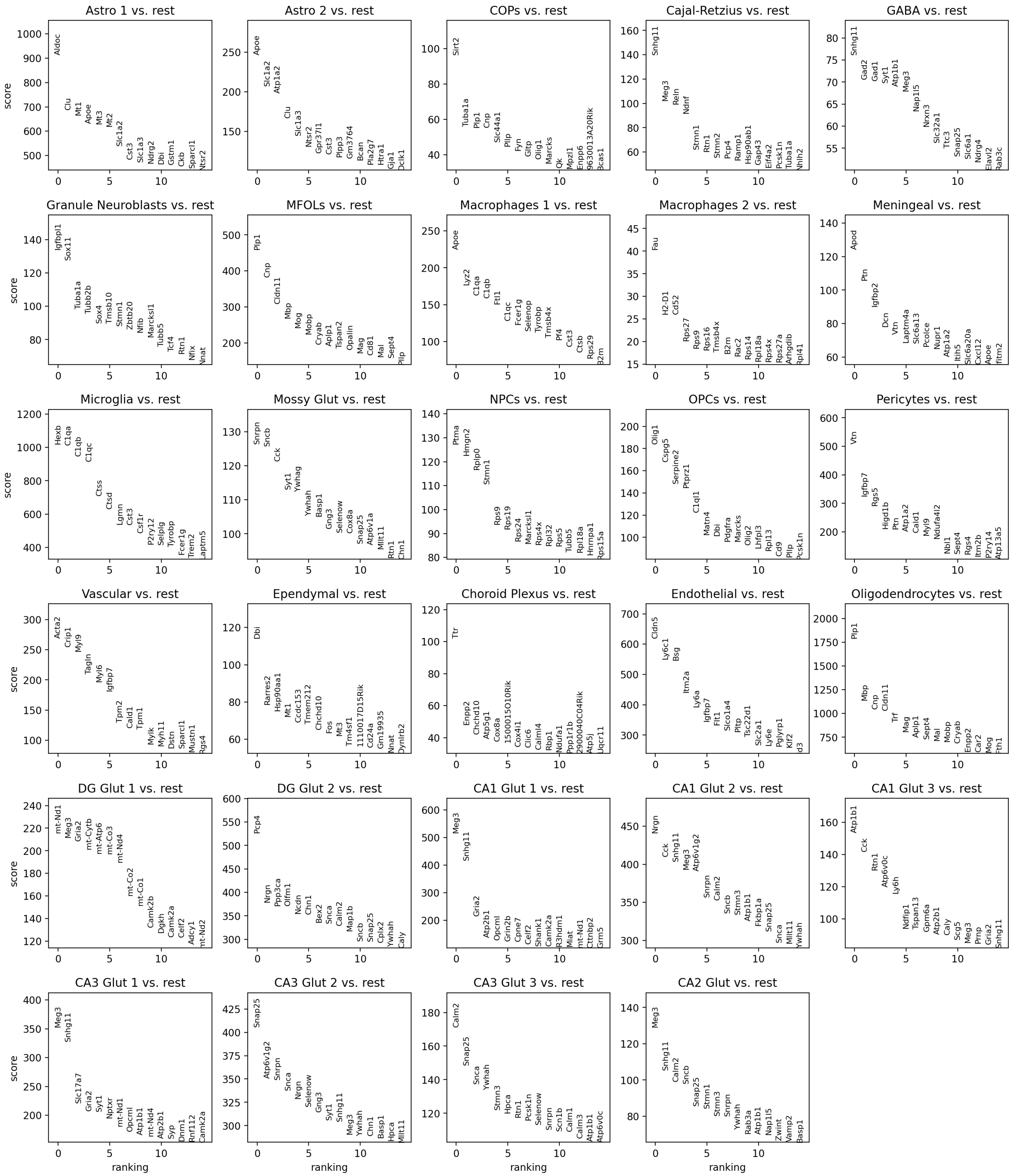

Supplemental Figure 1 (supplement to Fig. 1): Gene markers for the annotated cell types in the dataset
Each plot displays the top 15 marker genes for each annotated cluster of cells in the dataset. These markers were used to annotate cell types and were obtained using a Wilcoxon rank group test.

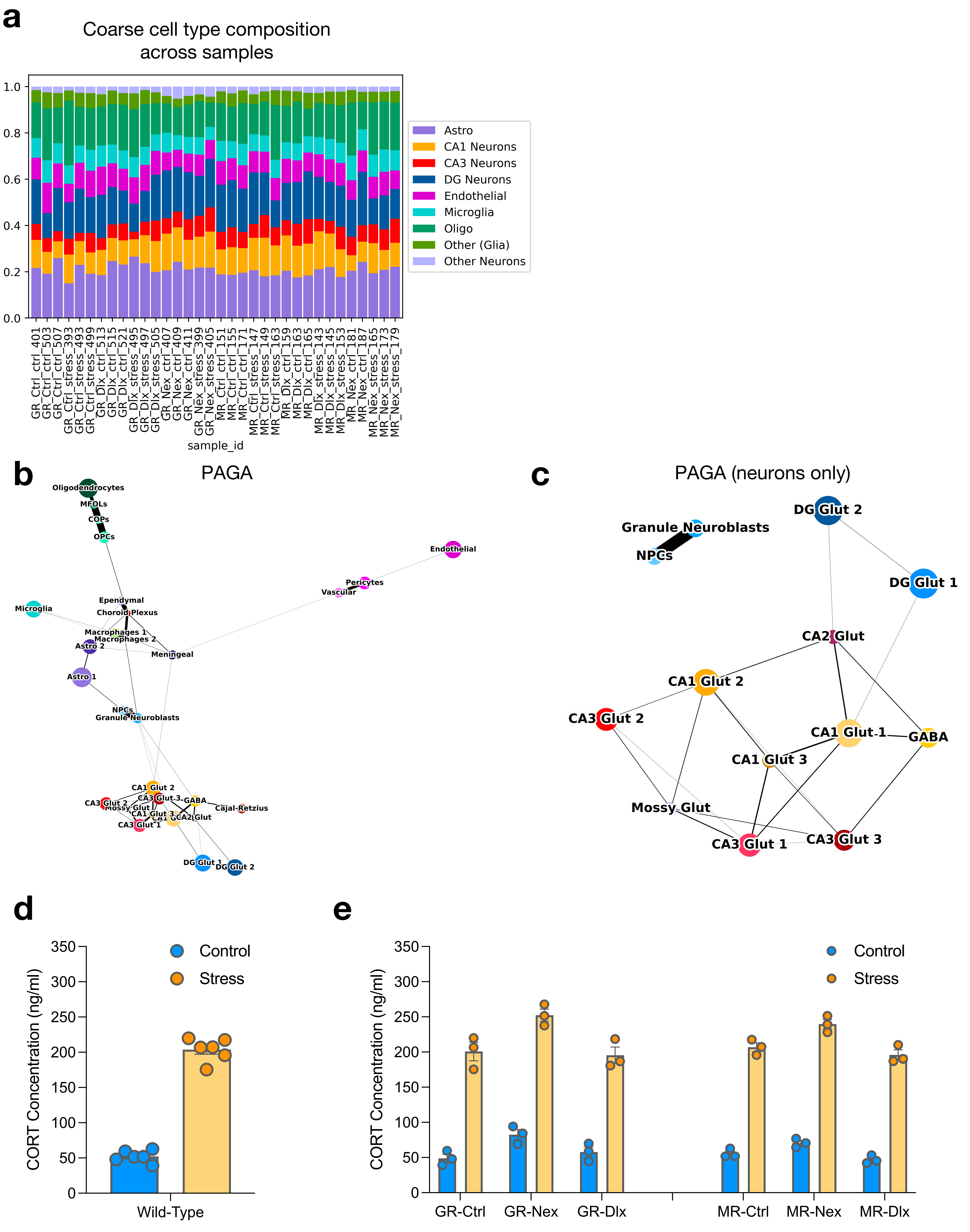

Supplemental Figure 2 (supplement to Fig. 1): Graph abstraction analysis of the single cell dataset and cell type composition
a) Coarse cell type composition across all samples in the dataset. **b)** Partition abstract graph analysis (PAGA) computed on the whole dataset and **c)** on neuronal cell types only.

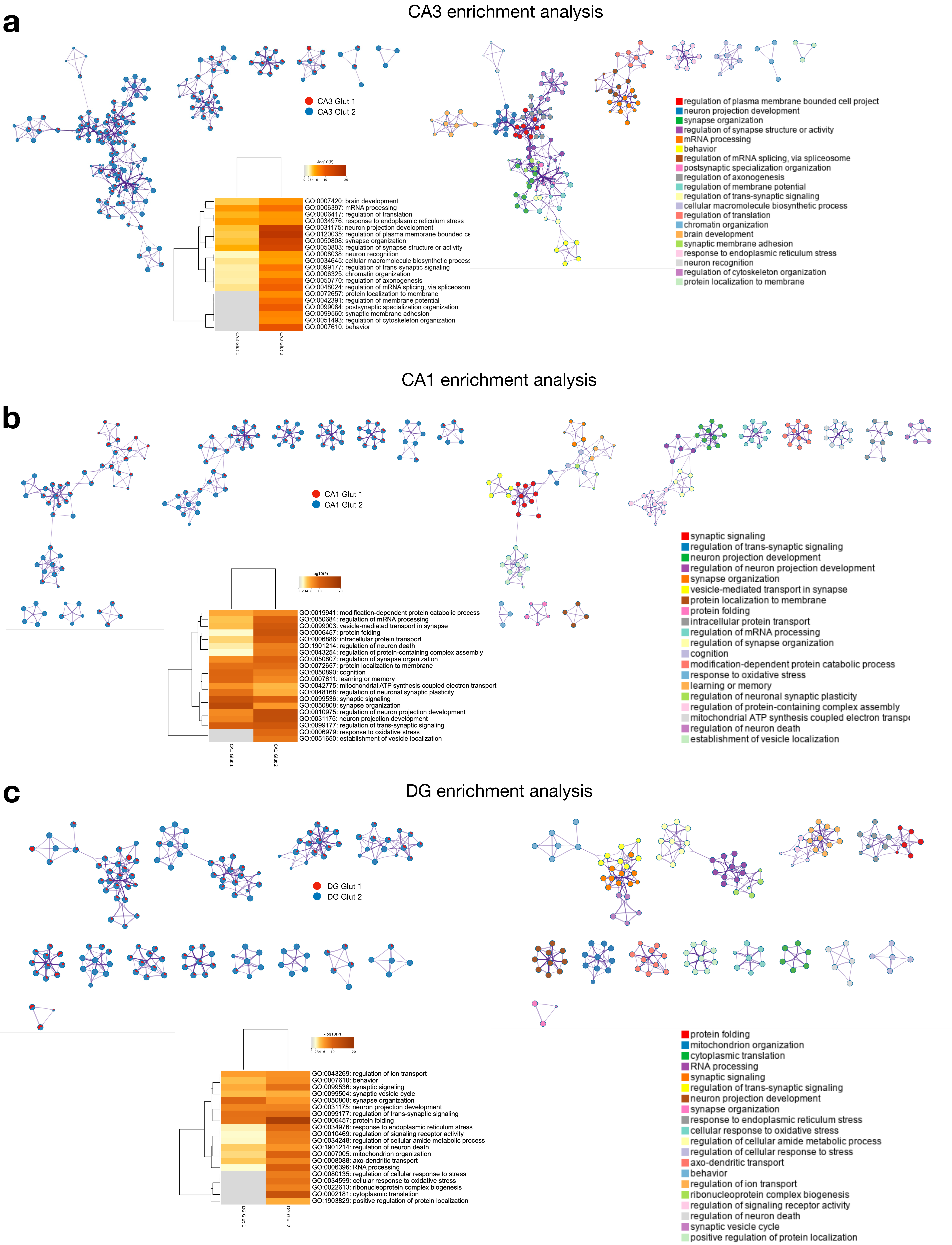

Supplemental Figure 3 (supplement to Fig. 2): Enrichment analysis on neuronal clusters
a) The graphs displays the biological process ontology terms that were found to be enriched in either CA3 Glut 1 and CA3 Glut 2 clusters. On the left nodes on the graph are shown as pie charts where each sector is proportional to the number of hits originated by the list of differentially expressed genes in a cell type. On the right, terms are represented by circle nodes, whose size is proportional to the number of input genes fall under that term, and whose color represent cluster identity. Terms with a similarity score > 0.3 are linked by an edge (the thickness of the edge represents the similarity score). The heatmap displays the p-value associated with biological processes in the two lists of genes tested. **b)** Analogous analysis for CA1 Glut 1 and CA1 Glut 2 clusters and **c)** for DG Glut 1 and DG Glut 2 clusters.

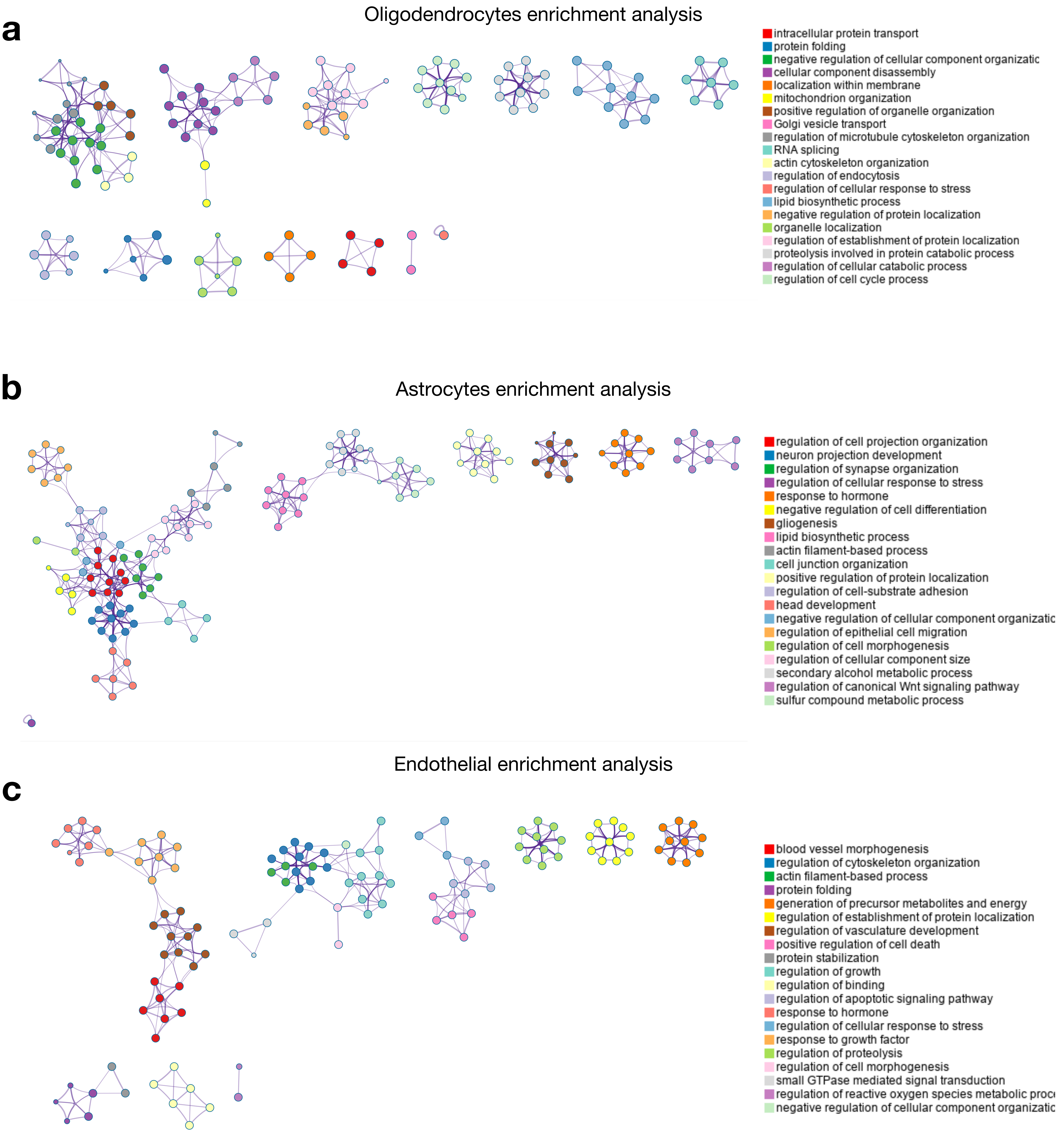

Supplemental Figure 4 (supplement to Fig. 2): Enrichment analysis on non-neuronal clusters
a) The graphs displays the biological process ontology terms that were found to be enriched in oligodendrocytes. Terms are represented by circle nodes, whose size is proportional to the number of input genes fall under that term, and whose color represent cluster identity. Terms with a similarity score > 0.3 are linked by an edge (the thickness of the edge represents the similarity score). **b)** Analogous analysis for Astro 1 and **c)** Endothelial cells.

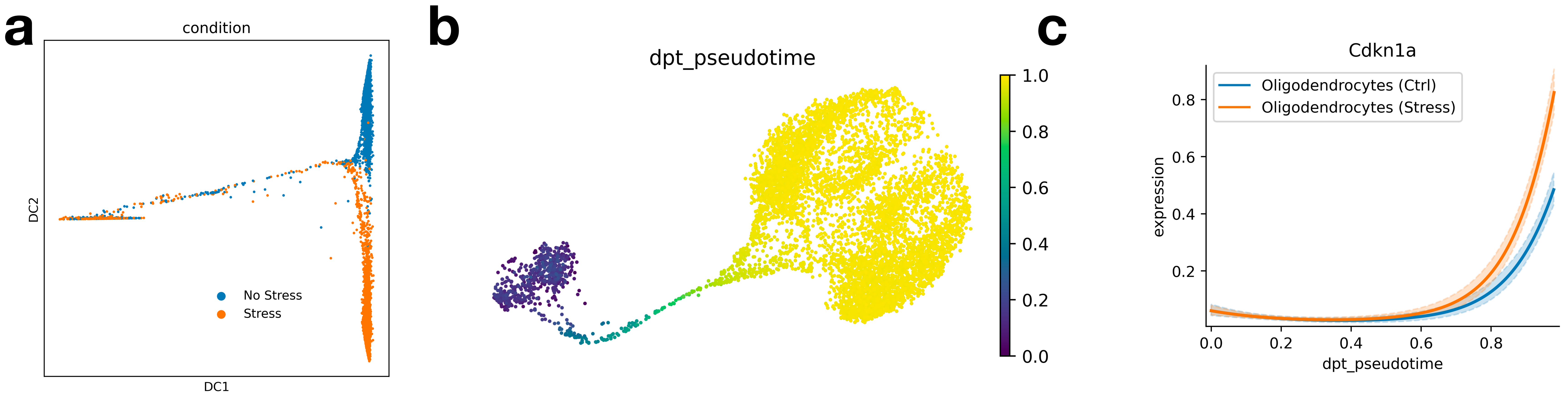

Supplemental Figure 5 (supplement to Fig. 3): Diffusion components and pseudotime analysis of oligodendrocytes in WT animals
a) Cells in the oligodendrocyte trajectory (OPCs - COPs - MFOLs - Oligodendrocytes) are shown along their first two diffusion components. These cells split by condition on the second diffusion component. **b)** Pseudotime obtained on diffusion components along the oligodendrocyte trajectory. **c)** *Cdkn1a* expression trends along the pseudotime covariate in control and stress conditions.

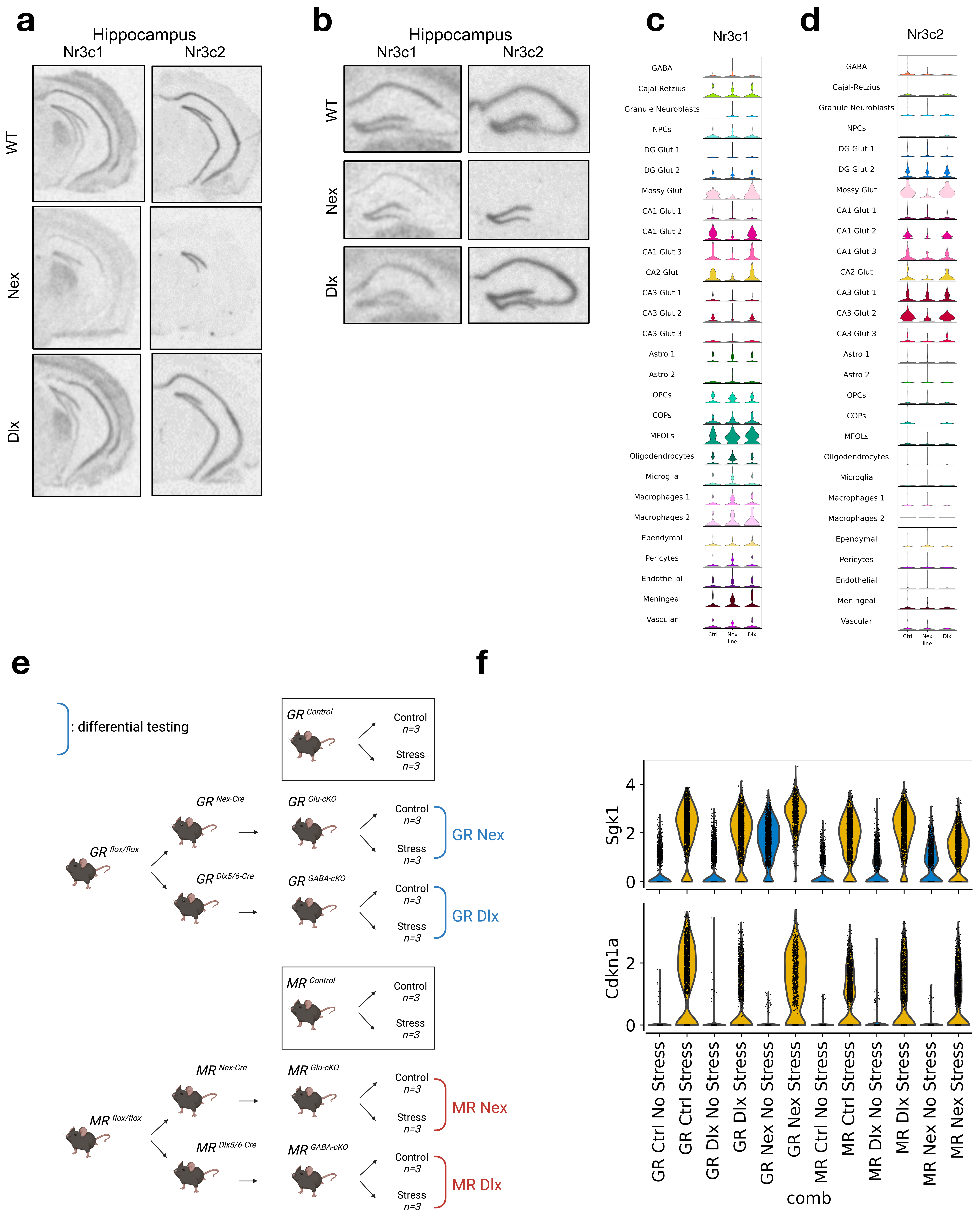

Supplemental Figure 6 (supplement to Fig. 4): Gene knockout expression, experimental design and *Sgk1* and *Cdkn1a* expression across KO lines. a-b) *In-situ* hybridization of *Nr3c1* and *Nr3c2* in the hippocampus of WT, Nex and Dlx mice. **c-d)** *Nr3c1* and *Nr3c2* expression across mouse lines and cell types. **e)** Differential tests performed and experimental design used to generate control and stress animals in knockout lines. **f)** *Sgk1* and *Cdkn1a* expression across all conditions including knockout lines. The effect of stress is consistent across conditions.

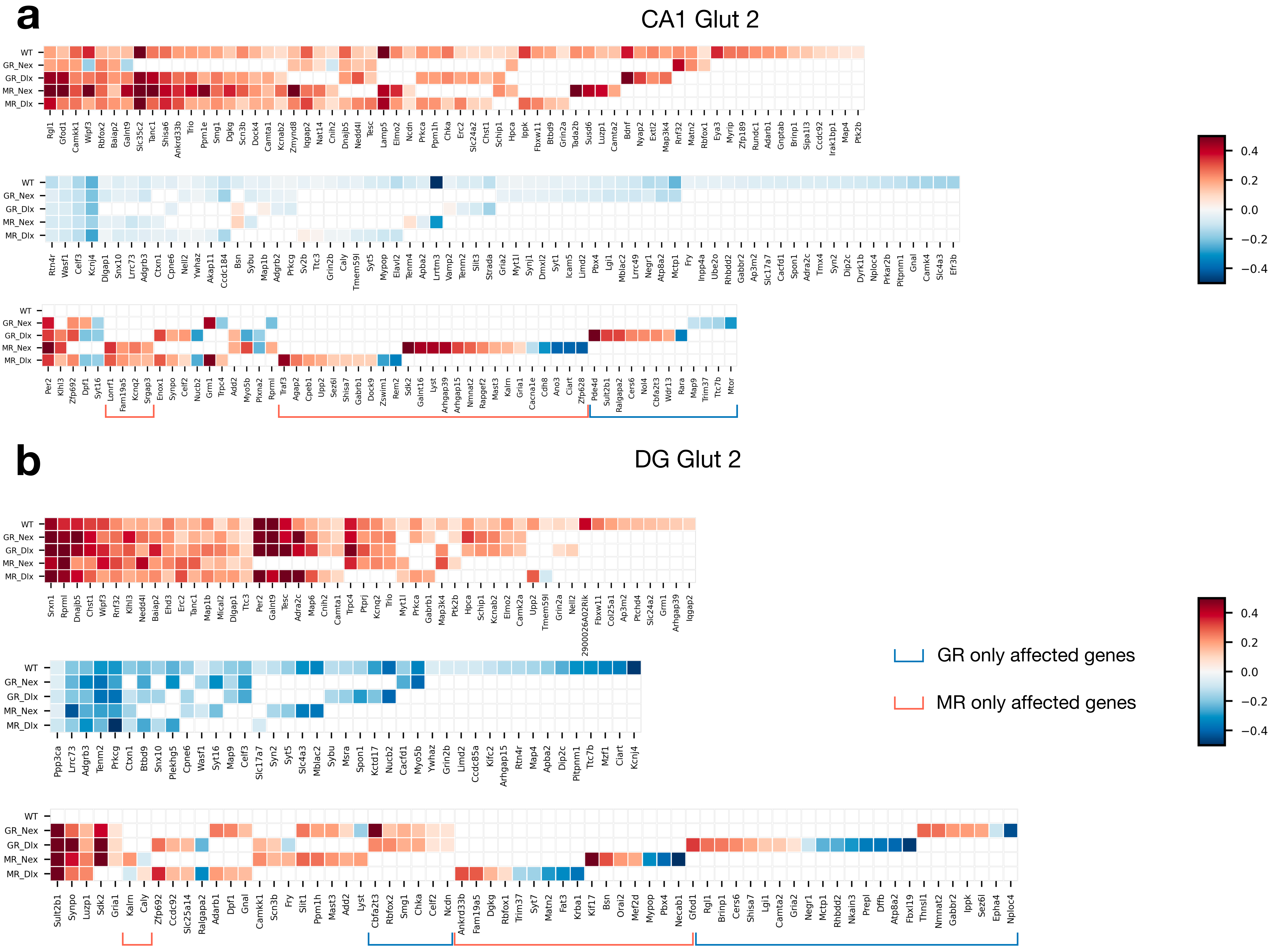

Supplemental Figure 7 (supplement to Fig. 5): Differential expression patterns of GR and MR targets across KO conditions
Heatmaps showing the Log2FC of known GR and MR targets (x-axis) in each tested condition (y-axis) in **(a)** CA1 Glut 2 and **(b)** DG Glut 2. The Log2FC is not shown in conditions in which the gene was not significantly differentially expressed. Only genes that were DE in at least one of the five conditions are displayed. Genes are separated between three groups: (top) genes that were upregulated in WT, (middle) genes that were downregulated in WT, (bottom) genes that were not significant in WT. With brackets we highlight genes whose expression is affected by either only GR or only MR knockouts. Log2FC: log2 fold change.

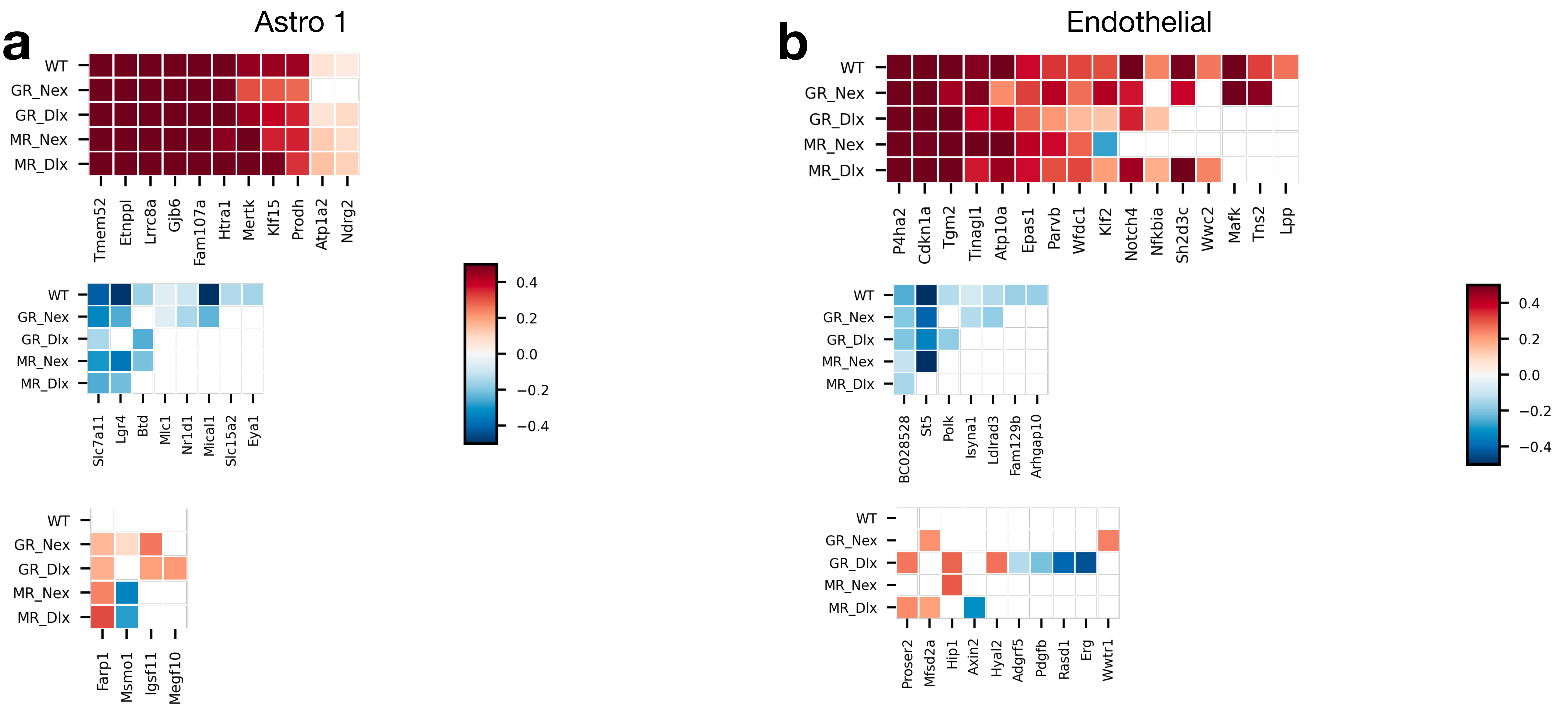

Supplemental Figure 8 (supplement to Fig. 5): Differential expression patterns of GR and MR targets across KO conditions
Heatmaps showing the Log2FC of known GR and MR targets (x-axis) in each tested condition (y-axis) in **(a)** Astro 1 and **(b)** Endothelial cells. The Log2FC is not shown in conditions in which the gene was not significantly differentially expressed. Only genes that were DE in at least one of the five conditions are displayed. Genes are separated between three groups: (top) genes that were upregulated in WT, (middle) genes that were downregulated in WT, (bottom) genes that were not significant in WT. With brackets we highlight genes whose expression is affected by either only GR or only MR knockouts. DE: differentially expressed.
